# A Network Analysis Approach toward Adaptive Overt Narcissism Network

**DOI:** 10.3390/bs13060468

**Published:** 2023-06-05

**Authors:** Remus Runcan, Dana Rad, Patricia Runcan, Cristian Măduța

**Affiliations:** 1 Center of Research Development and Innovation in Psychology, Faculty of Educational Sciences Psychology and Social Work, Aurel Vlaicu University of Arad, 310032 Arad, Romania; 2 Department of Social Work, Faculty of Sociology and Psychology, West University of Timişoara, 300223 Timișoara, Romania; 3 Faculty of Humanities and Social Sciences, Aurel Vlaicu University of Arad, 310032 Arad, Romania

**Keywords:** grandiose, vulnerable, art, network analysis, expected influence

## Abstract

The relationship between narcissistic personality and art and beauty appreciation has recently become the focus of research investigations. Adaptive narcissists raise their sense of worth in order to shield themselves from harm caused by others. Because they aspire to be more attractive, healthier, and successful versions of themselves, they frequently have greater success in life than the majority of people. Grandiose and overtly narcissistic behavior are the main recognized characteristics of an overt narcissist, which is currently regarded as a personality disorder that puts mental health and wellbeing at peril. On a random sample collection of data from 1101 respondents to an online questionnaire, we conducted a network analysis of the Adaptive Overt Narcissism Scale (AONS) items. In this study, we used a network analysis approach to examine the network structure of adaptive overt narcissism, as well as its relationships with psychological functioning. The present study utilized network analysis to investigate the centrality measures of items in the Adaptive Overt Narcissism Scale (AONS) and their interrelationships. Results indicated that item Q6.8 (“I appreciate art and beauty”) had low betweenness, closeness, and strength centrality measures, indicating that it was less influential in the network. However, it also had negative expected influence, suggesting that its absence would have a destabilizing effect on the network. These results highlight the importance of the appreciation of art and beauty in deactivating the adaptive overt narcissist network. Further research is needed to explore the mechanisms underlying this relationship and its implications for narcissism prevention and intervention.

## 1. Introduction

Narcissism is a complex personality trait characterized by grandiosity, entitlement, and a lack of empathy. While traditionally viewed as a unidimensional construct, recent research has suggested that narcissism may be better conceptualized as a multidimensional construct, with distinct facets such as overt and covert narcissism. Overt narcissism is characterized by exhibitionism, dominance, and a need for admiration, and it is associated with a range of negative outcomes, including aggression, poor interpersonal relationships, and lower psychological wellbeing.

To provide a comprehensive understanding of narcissism, it is important to consider contemporary perspectives. Modern research suggests that narcissism can be viewed as a relatively “light” trait within the dark triad, as it often does not entail a decrease in intellectual functioning. Furthermore, recent findings indicate that narcissists may not necessarily have low empathy but may prioritize other aspects over manifesting empathy, emphasizing the nuanced nature of this trait [1,2].

Despite its importance, the nature and structure of overt narcissism remain poorly understood. In particular, little is known about the patterns of co-occurrence and interdependence among the different components of overt narcissism. Network analysis provides a powerful framework for investigating such complex relationships among variables. By representing overt narcissism as a network of interconnected components, we can examine the dynamic interplay among these components and gain insights into the structure of the construct.

In this paper, we use a network analysis approach to investigate the network structure of overt narcissism. Specifically, we focus on adaptive overt narcissism, which refers to the positive aspects of overt narcissism that may facilitate success and wellbeing. We aim to identify the key components of adaptive overt narcissism, examine the patterns of co-occurrence and interdependence among these components, and explore their implications for psychological functioning.

Additionally, we aim to examine the influence of art and the appreciation of beauty on the networks of overt narcissism, hypothesizing that higher levels of engagement with art and beauty will weaken the connections among the components of overt narcissism. This deactivation of narcissistic networks is expected to lead to reduced levels of narcissistic behavior and attitudes, fostering improved interpersonal relationships, enhanced wellbeing, and increased empathic tendencies. By elucidating the complex interplay among the components of overt narcissism and exploring the potential influence of art and beauty, this study contributes to our understanding of the structure and adaptive aspects of narcissism. Furthermore, it may inform interventions targeting individuals high in overt narcissism, highlighting the potential benefits of engaging with art and promoting the appreciation of beauty.

## 2. Literature Review on Narcissism

The concept of narcissism has been of interest to researchers across a range of disciplines for many years, and it has been investigated from various perspectives. However, in the last two decades, the phenomenon of narcissism has become increasingly intertwined with social media use. Indeed, recent research has suggested that social media platforms such as Facebook may facilitate the expression and validation of narcissistic traits [3,4,5].

One study highlighted the relationship between narcissism and social media use [3]. The authors argued that narcissistic individuals are more likely to engage in self-promotion on Facebook, and that the pursuit of likes and positive feedback on the platform may serve to reinforce and amplify these tendencies. In other words, Facebook may provide a fertile environment for the development and expression of narcissistic traits.

This notion is supported by a growing body of research on the topic. For example, studies have found that individuals high in narcissism tend to post more self-promoting content on social media [4], use more first-person singular pronouns in their posts [5], and are more likely to engage in social comparison and envy-inducing behavior on these platforms [1,2]. Additionally, some studies have suggested that the use of social media may contribute to the development of narcissistic tendencies in some individuals [6,7,8,9,10,11,12,13,14,15,16,17].

Despite the growing interest in the relationship between narcissism and social media use, it is important to note that the construct of narcissism itself has a long and complex history in the psychological literature. Narcissism has been measured using a range of different scales and instruments, including the Narcissistic Personality Inventory (NPI) [18,19], the Narcissistic Personality Disorder Scale (NPDS) [20], and the Minnesota Multiphasic Personality Inventory (MMPI) [21,22,23]. These instruments typically assess various aspects of the construct, such as grandiosity, entitlement, and exhibitionism.

One of the earliest and most widely used measures of narcissism is the Murray’s Narcissism Scale [17]. This scale, developed by Henry Murray in the 1930s, assesses various dimensions of narcissism, including self-esteem, exhibitionism, and autonomy. The scale was initially developed for use in clinical settings, but has since been used in a range of research contexts.

Another commonly used measure of narcissism is the NPI, developed by Raskin and Terry in 1988 [18]. This 40-item questionnaire assesses various aspects of narcissism, such as a sense of entitlement, a need for admiration, and a lack of empathy. The NPI has been used in a wide range of research contexts, and it has been found to be a reliable and valid measure of narcissism [19].

The NPDS, developed by Wink in 1991, is a 34-item self-report measure designed to assess the symptoms of narcissistic personality disorder [20]. The scale assesses a range of narcissistic traits, including grandiosity, entitlement, and a lack of empathy. The scale has been used in both clinical and nonclinical settings, and it has been found to be a reliable and valid measure of narcissistic personality disorder.

The MMPI, first developed in the 1930s, is a widely used personality assessment tool that includes a range of scales designed to assess various personality traits and psychological disorders [22,23]. The MMPI has been used in numerous research contexts, and it includes a Narcissistic Personality Inventory scale that assesses grandiosity, entitlement, and a need for admiration.

Despite the availability of these various scales and measures of narcissism, it is important to note that the construct itself is not without controversy. Some researchers have argued that the construct of narcissism is poorly defined, and that different measures may capture different aspects of the phenomenon [24]. Others have pointed out that narcissism may be a complex and multifaceted construct, with different subtypes or dimensions [25,26]. Indeed, some have suggested that there may be important distinctions to be made between overt and covert forms of narcissism [27,28].

Given the complexity and diversity of the construct of narcissism, it is perhaps not surprising that there is ongoing debate and discussion in the literature about how best to measure and conceptualize the phenomenon. However, it is clear that narcissism is a construct of considerable interest to researchers and practitioners alike, and that social media use may be an important context in which to investigate its expression and impact.

In particular, the notion that social media platforms may serve to amplify and reinforce narcissistic tendencies has important implications for our understanding of how these platforms are used and experienced. For example, research has suggested that social media use may contribute to the development of a “selfie culture”, in which individuals are increasingly focused on presenting and promoting themselves online [29,30]. Similarly, social media may contribute to the rise of influencer culture, in which individuals with large followings use their platforms to promote products and lifestyles in ways that may be perceived as narcissistic [31].

Of course, it is important to note that not all individuals who use social media are narcissistic, and that many individuals use these platforms in healthy and positive ways. However, the relationship between social media use and narcissism is an area of growing interest and concern, and one that requires further investigation and discussion.

Narcissism has been a subject of study for several decades, with researchers exploring various facets of this personality trait. One of the areas of interest has been the identification and differentiation of two forms of narcissism: overt and covert [23,24]. These two forms of narcissism are distinguished by their expression of different behaviors, attitudes, and feelings.

Overt narcissism is characterized by a grandiose sense of self-importance, arrogance, and exhibitionism [25]. Individuals with this form of narcissism are often preoccupied with receiving admiration and attention from others and have a flagrant display of superiority. They exhibit a direct expression of their exhibitionism and lack empathy for others. Overt narcissism is associated with exploitative tendencies and a sense of entitlement [26].

On the other hand, covert narcissism is characterized by feelings of grandeur that are largely unconscious, a lack of zest for work, and a lack of self-confidence. Individuals with covert narcissism have vague feelings of depression, anxiety, grandiose fantasies, hypersensitivity, and insecurity. They also exhibit exploitative tendencies and a sense of entitlement [26].

To further explore these two forms of narcissism, researchers have used a range of measures, including the Profile of Narcissistic Dispositions (POND) and Kohut measures of grandiosity and idealization [26,27,28,29,30,31,32]. These measures have been used to identify three clusters of narcissism: overt, adaptive, and covert. The overt narcissism group is characterized by high scores on all of the POND dimensions, medium scores on the pathology of separation/individuation, and low scores on idealization and grandiosity. This group displays a pattern of behavior consistent with the classic symptoms of overt narcissism, including a grandiose sense of self-importance, arrogance, and a flagrant display of superiority [26,27,28,29,30,31,32]. The adaptive narcissism group, on the other hand, reports a range of low, high, and medium scores on each of the narcissism measures, with the pathology of separation/individuation scale having the lowest mean score. This group displays a more balanced profile of narcissistic traits, with some evidence of healthy narcissism [26,27,28,29,30,31,32,33,34,35]. Lastly, the covert narcissism group reports a profile of low to medium scores on all narcissistic measures, except for the pathology of separation/individuation, which was reported at a medium level. This group exhibits a pattern of behavior consistent with the classic symptoms of covert narcissism, including feelings of grandeur that are largely unconscious, a lack of zest for work, and a lack of self-confidence [26,27,28,29,30,31,32,33,34,35,36].

It is worth noting that the distinction between overt and covert narcissism is not always clear-cut, and there may be some overlap between the two forms [36]. Additionally, there may be subtypes or dimensions of narcissism beyond these two forms that warrant further investigation [36].

Overall, the identification and differentiation of overt and covert narcissism is an important area of research, as it helps us better understand the complex and multifaceted nature of this personality trait. By using a range of measures and exploring different clusters of narcissism, researchers can gain a more nuanced understanding of how narcissism is expressed and experienced. This knowledge can be useful for developing interventions and treatments for individuals who struggle with narcissistic tendencies, as well as for informing broader discussions about the role of narcissism in contemporary society.

In conclusion, the construct of narcissism has a long and complex history in the psychological literature, and it has been investigated from a range of perspectives and using a variety of measures. Recent research has suggested that social media use may be an important context in which to investigate the expression and impact of narcissistic tendencies. As such, understanding the relationship between narcissism and social media use has important implications for our understanding of how these platforms are used and experienced, and for our broader understanding of the nature of the self in contemporary society. Further research in this area is warranted, in order to better understand the complexities of this relationship and its implications for individuals and society as a whole.

### Narcissism and Art and Beauty Appreciation

In a critical analysis of the concept of narcissism, previous research [37] conducted an examination and revision of the assumptions associated with this construct. The study challenged the notion that opposition to prevailing social forms represents a regression to narcissism. This assumption has been widely accepted in the literature. The study suggested that this assumption warrants serious consideration due to its prevalence. It was argued that alternative forms such as myth and art, which surpass societal structures, may be perceived as manifestations of narcissism. Consequently, these alternative forms were proposed as potential substitutes for the existing social order, albeit characterized by narcissistic tendencies.

In a separate study, the authors of [38] argued and provided empirical evidence to support the notion that Machiavellianism, which involves the manipulation of others, disdain for conventional morality, and viewing humankind with cynicism, is a theoretically appreciable personality configuration that is distinct from narcissism. The authors also posited that certain components of perfectionism form part of this configuration. Through their analysis, they sought to provide a nuanced understanding of the various personality traits and configurations that contribute to the construct of narcissism.

In one study [7], it was posited that narcissists manipulate others as part of their pathology by interacting “with their own reflection in the mirror of your face and actions”. They “pose”, i.e., adjusting their image by how they act and carve out a work of art. Similarly, the authors of [39,40] found that grandiose narcissistic individuals exhibit self-protective behaviors, such as derogation or devaluation, when threatened by comparison with a better-performing other or by negative feedback. These individuals also self-report concerns for self-presentation, status, power, dominance, and physical beauty.

In terms of generational differences, the authors of [39] suggested that Millennials resemble adults of other generations in many of their lifestyle choices due to their ideology or socioeconomic status, rather than their age. While they may be in the mainstream when it comes to areas such as going green and gun ownership, they express their uniqueness through other means such as body art and technology. Regarding creativity in teens, the authors of [41] pointed out that social media and mobile internet usage allows for creative expression through the sharing and remixing of online content such as artwork, photos, stories, and videos. Furthermore, the authors of [9] highlighted the expression of grandiosity in narcissistic individuals through unwarranted expectations, exceptionally high aspirations, and self-centeredness, as well as in fantasies of unfulfilled ambitions or unlimited success, power, brilliance, beauty, or ideal relationships.

According to the authors of [42], individuals who exhibit narcissistic traits, such as vanity, exhibitionism, superiority, and entitlement, and those who demand perfection of themselves tend to show interest in cosmetic surgery as they search for an objectively successful and aesthetically pleasing outcome. The authors of [33] noted that typical narcissistic children often have fantasies of wealth, power, beauty, or accomplishment. The Adaptive Overt Narcissism Scale (AONS), as validated in [28], includes an item on the appreciation of art and beauty.

A narcissistic personality, as defined in [43], is preoccupied with fantasies of unlimited success, power, brilliance, beauty, or ideal love. The authors of [31] made correlations between the Narcissistic Personality Inventory (NPI-10 and NPI-20) and the Hypersensitive Narcissism Scale (HSNS). The authors of [44] argued that women use selfies to move away from the idealized version of themselves and toward a more realistic representation, while warning that excessive selfie-taking does not necessarily correlate with body image disorders.

As stated in [45], individuals high in narcissistic traits are often charming, talented, successful, beautiful, and charismatic, and are able to cast a spell on others through compliments, scintillating conversation, and apparent interest in others. Lastly, the authors of [46] suggested that individuals high in openness (extraverts) are more likely to share information about intellectual topics, such as art, writing, research, current events, politics, and science, on social media platforms such as Facebook.

In one study [47], the motives for using Instagram were examined, including their relationship with contextual age and narcissism. The study found that “creativity”, including items such as “to find people with whom I have common interests”, “to show off my photography skills”, and “to create art”, was one of the four primary reasons for using this social networking service. Additionally, individuals who performed well in interpersonal connection tended to use Instagram more frequently for coolness, creativity, and surveillance. According to [48], individuals with covert narcissism have a grandiose sense of self, preoccupations with fantasies of power, and a requirement for excessive admiration. However, they may hide these attributes to gain the trust and liking of others. The authors of [49] suggested that the reason for the relationship between selfies and narcissism is that selfies generate strong emotions. Every narcissist requires a reflecting pool, and social networking sites such as Facebook have become our modern-day pool. Previous studies such as [38,40,42] confirmed the findings of [50], indicating that self-oriented perfectionism is not just an extreme need for achievement, but may also involve a willingness to exploit others in the pursuit of status, power, dominance, and physical beauty.

Our hypothesis is motivated by recent research on psychopathology symptom networks [51], which suggests that more connectedness in symptom networks is associated with more severe psychopathology. We hypothesize that art and beauty appreciation weaken the connectivity in overt narcissism networks. We investigate this statement by analyzing the effect on the item referring to art and beauty appreciation on the remainder of the scale’s items, in terms of expected influence. In order to depict a network deactivation effect of the art and beauty appreciation over the overt narcissism network, the expected influence sign has to be negative, and the overall weight coefficients must be high in rank.

Specifically, we hypothesize that higher levels of engagement with art and the appreciation of beauty will weaken the connections among the components of overt narcissism, leading to reduced levels of narcissistic behavior and attitudes. This negative influence can be understood as a potential reduction in self-centeredness, grandiosity, and entitlement, resulting in enhanced interpersonal relationships, improved wellbeing, and increased empathic tendencies. By deactivating the networks of narcissism through the evaluation of art and beauty, individuals may experience a psychological shift characterized by greater self-reflection, empathy, and a more balanced perspective. These effects are expected to contribute to personal growth, enhanced emotional regulation, and the development of healthier social connections.

Regarding the network analysis results, we expect that the art and beauty appreciation item would have the highest negative expected influence over the overt narcissism scale network. This result would prove the deactivation effect that art and beauty appreciation have over the general recognized symptoms of overt narcissism.

The investigation of the adaptive overt narcissism network is a relevant and valuable research topic for several reasons. Firstly, overt narcissism is a personality trait associated with a range of negative outcomes, including relationship problems, low empathy, and difficulty handling criticism. However, recent research has highlighted that there may be a more positive, adaptive side to narcissism, which includes traits such as confidence, self-esteem, and assertiveness. By investigating the network of traits associated with adaptive overt narcissism, researchers can gain a deeper understanding of how these adaptive traits interact and contribute to overall personality functioning.

Secondly, a network analysis approach is a novel and innovative way of investigating personality traits. This approach views personality traits as interconnected nodes within a larger network, rather than as isolated characteristics. By examining the network of traits associated with adaptive overt narcissism, researchers can gain a more nuanced understanding of the complex interplay between different traits and how they influence overall personality functioning.

Thirdly, understanding adaptive overt narcissism has important practical implications. Individuals with high levels of overt narcissism can benefit from interventions that focus on enhancing their adaptive traits while mitigating the negative outcomes associated with maladaptive narcissism. By identifying the network of traits associated with adaptive overt narcissism, researchers can develop targeted interventions that focus on enhancing the adaptive traits while minimizing the negative ones.

In conclusion, investigating the network of traits associated with adaptive overt narcissism is a worthwhile and valuable research topic. By using a network analysis approach, researchers can gain a deeper understanding of the complex interplay between different traits and how they contribute to overall personality functioning. This understanding has important practical implications for the development of targeted interventions for individuals with high levels of overt narcissism.

## 3. Materials and Methods

### 3.1. Network Analysis Methodology

Academics can use network analysis to depict operator relationships and investigate the social structures that emerge as a result of the recurrence of these links [51]. The core premise is that we may better understand how items interact with one another and the overall item structure by understanding the connections between the items on a scale. When items are represented as nodes and their interactions are represented as lines connecting pairs of nodes, the concept of a social network changes into a useful analytical tool utilizing the mathematical language of graph theory [51].

In contrast to the majority of other quantitative social science areas, statistical issues have received very little attention in network analysis. Without taking into consideration sample variation, measurement error, or other unknowable variables, the majority of methodologies and metrics analyze the structure of particular datasets. Due to the reliance present in network data, these challenges are complicated, yet they are becoming more prevalent.

The structure of psychological items may be investigated using a modern approach called psychometric network analysis [51]. Although the assessment of constructs such as dimensional structure, for instance, has dominated the psychometric network literature to far, there are other uses for psychometrics. In this study, we investigate the potential of network analysis in discovering what particular item triggers the deactivation of the overt narcissistic psychological profile within the framework of the Adaptive Overt Narcissism Scale (AONS).

A technique for looking at the network structure of variables is network analysis. Our network analysis must be a sufficiently accurate representation of the underlying data for us to guarantee its scientific accuracy [52]. This research is mainly focused on network analysis to study the deactivation mechanism of overt narcissism, by looking at the relationship among Adaptive Overt Narcissism Scale (AONS) items.

In the context of this research, a network is a collection of structures that consists of nodes, which act as placeholders for variables, and edges, which act as links connecting these nodes. The centrality and expected influence indices seek to exploit the intricate network’s structure to pinpoint symptoms that ought to be particularly crucial to its growth and durability (in our case, overt narcissism).

According to the authors of [51], a node’s connections make up the anticipated influence, which measures a node’s relative relevance in a network. Even in networks with weak connections, this node will always have a significant predicted effect in the case of standardized outcomes (with generally low edge weights). The psychopathology network approach sees mental illnesses as networks of linked nodes that reinforce one another, e.g., symptoms (in our case, items of the overt narcissism scale). Researchers that have taken this approach have proposed that network topology may be used to pinpoint key nodes that affect the disease, with the onset and maintenance of the disorder being most affected by nodes near the network’s core. In contrast, frequently used centrality indices do not differentiate between positive and negative edges, making it impossible to predict the kind and extent of a node’s influence inside the network. To overcome this drawback, the authors of [51] created two expected influence (EI) indices for nodes that take into account the existence of negative edges. To better identify highly significant nodes in mental disease networks, it may be required to discriminate between positive and negative edges. Two additional node important methods are suggested to mitigate this risk. In contrast to measures of centrality, which quantify a node’s position within a network, these indices try to investigate the nature and strength of a node’s cumulative effect inside the network and, hence, the role it is expected to play in the network’s activation, persistence, and remission.

We calculated three indices of node centrality using the R package qgraph [53,54,55,56,57,58]: node strength, closeness, and betweenness. Higher values for each centrality measure indicate more network centrality. The predicted influence was then calculated. If a node is anticipated to have a positive impact, changes to the node should result in changes to the entire network in the same way. If the anticipated effect of a node is negative, changes in the node should cause network changes in the opposite direction [51].

### 3.2. Participants

The participants in this research were recruited using a convenience sampling technique, as the research was a cross-sectional investigation aiming to depict the expected influence of art and beauty appreciation in the deactivation of adaptive overt narcissism network. After obtaining ethical approval for the investigation, an online survey was created and shared on social media public networks. A total of 1101 anonymous, valid, and consented responses were received from Romanian participants.

In terms of gender, 37% of the participants were male respondents and 63% represented female respondents. Furthermore, 75% of the participants came from urban areas, while 25% were from rural areas. Age-wise, 64% of the respondents were aged between 18 and 35 years old, 23% were aged between 36 and 50 years old, and 13% were aged over 51 years old. Regarding education, 41% of the participants had graduated high school, 6% had completed post-secondary school, 43% had finished higher education studies, and 10% were holders of a PhD degree. In terms of marital status, 55% were single or not married, 40% were married, and 5% were divorced or widowed.

### 3.3. Instrument

The Adaptive Overt Narcissism Scale (AONS) was validated by [28]. Their research was carried out on 420 female and male adults an on 175 college women. The 20 items of the new AONS [29] were adapted from three sources: items 1–10 and 14–20 from [25]; items 11 and 12 from [59]; item 13 from [17]. The items of Murray’s Narcissism Scale [17] and a composite measure of covert narcissism based on the Minnesota Multiphasic Personality Inventory (MMPI) [22,23] were correlated to derive a new measure of hypersensitive narcissism—the Hypersensitive Narcissism Scale (HSNS), containing 10 items [60].

AONS has 20 items; answers are scored on a scale of 1–5: 1 = not at all, 2 = very little, 3 = neutral, 4 = very much, 5 = extremely.

In items 9 and 10, the value of the statements is overturned, which is offered by the authors as a way of interpreting the results, with a higher score representing a greater presence of narcissism in the respondent person.

A score above 80 points represents a very high level of narcissism, a score of 60–79 points represents an average level of narcissism, and a score of 40–59 points represents a weak level of narcissism. Below 40 points, one cannot speak of narcissism.

## 4. Results

### 4.1. Descriptive Statistics

As the only instrument used in this research was the Adaptive Overt Narcissism Scale (AONS), in Table 1, we present the descriptive statistics in terms of meads, standard deviations, and Pearson correlation coefficients for each of the 20 items with item Q6.8. “I appreciate art and beauty”.

As depicted in Table 1, means for the 20-item scale ranged from 2.972 for individualistic traits to 4.009 for art and beauty appreciation. The overall scale mean was 3.61, representing a median score, i.e., an average level of narcissism.

In terms of reliability statistics, the scale obtained a Bartlett’s test χ² of 8285.291 at *p* < 0.001, a chi-squared test value of 515.191 at *p* < 0.001, and a Cronbach’s α coefficient of 0.873. In terms of additional fit indices, the scale yielded an RMSEA of 0.061 and a TLI of 0.902, all results indicating a valid and reliable measure of overt narcissism.

### 4.2. Network Analysis Results

Our hypotheses were inspired by recent findings [51,61,62,63,64,65] from psychopathology symptom networks. The findings imply that more severe psychopathology is associated with symptom networks with greater connectedness.

We hypothesize that art and beauty appreciation weakens the connectivity in adaptive overt narcissism networks. We investigate this statement by analyzing the effect on the item referring to art and beauty appreciation on the remainder of the scale’s items, in terms of expected influence. In order to depict a network deactivation effect of the art and beauty appreciation over the adaptive overt narcissism network, the expected influence sign has to be negative, and the overall weight coefficients must be high in rank.

Regarding the network analysis results, we expected that art and beauty appreciation item would have the highest negative expected influence over the adaptive overt narcissism scale network. This result would prove the deactivation effect that art and beauty appreciation has over the general recognized symptoms of overt narcissism.

A Gaussian graphical model (GGM), a network whose edges indicate partial correlations of ordinal or continuous data, was used to estimate the overall adaptive overt narcissism network in the pooled sample (using Jasp 0.13.3.0 and R packages qgraph and glasso). In order to conduct unbiased centrality analysis, partial correlations guarantee that interactions among nodes are not masked by associations with other network variables. The graphical lasso, a technique that uses regularization to prevent the estimation of spurious edges, is typically used to estimate GGMs. As a consequence, networks that are sparser and easier to understand are established, in which covariance between nodes is explained using only the minimal number of edges. After accounting for all other network factors, two nodes are considered to be statistically linked if an edge links them together in the resultant graph. They are conditionally independent if no edges exist.

Centrality measurements were performed to examine which components were most important to networks. High-centrality nodes have strong links to many other nodes, whereas low-centrality nodes are on the periphery of the network with fewer and poorer connections. Understanding how nodes are connected allows us to determine how clinically significant each node is. As a consequence, node strength and predictability were chosen as the centrality measures under consideration.

Node strength, which offers a relative measure of centrality, is the total of all edges connected to the node. The predicted effect of a node shows the shared variance of each node with its neighbors and is an absolute measure of connection.

For the network analysis, we used JASP version 0.16.3.0 and additional R packages. The overall network was designed with the 20 items of the AONS scale, represented by the 20 nodes. The number of nonzero edges was 121 from the total of 190 edges. The sparsity index [66,67] is a summary metric based on node counts, node degrees, and a constant factor that is at least as large as the sum of all node degrees in the network. It gives a hint as to the degree of degree distribution heterogeneity among the network’s individual nodes. The overall sparsity coefficient of the AONS network was 0.363.

**Figure 1:**
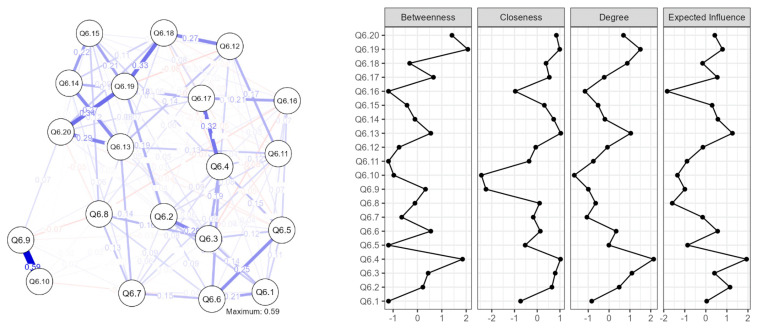
Network architecture.

The whole sample’s estimated network is shown in Figure 1 (left). AONS items are portrayed by each node, while connections between items are represented by edges, controlling for all other parameters. Positive edges were present on every node in the global network, and larger edges indicate greater relationships between nodes.

The lines and circles denote the variables and edges, respectively, while the thickness and blackness of the lines denote the weights of the edges. Positive relationships are shown by the blue edges, while negative associations are indicated by the red edges.

The strongest edges were between item 9 (I tend to be submissive, more of a follower than a leader (R)) and item 10 (I give up or even withdraw in the face of frustration and adversity (R)), both used with reverse scoring (0.59). Thus, the strongest association was between items reflecting dominance and resilience, as single items in the adaptive overt narcissism scale.

Moderate edges were shown between item 19 (I am ambitious) and item 20 (I am self-confident) (0.34), as well as between item 17 (I am clever) and item 4 (I have a high degree of intellectual capacity) (0.32).

Figure 1 (right) and Table 2 present the centrality metrics for the overall AONS network.

The measures included betweenness, closeness, strength, and expected influence.

Betweenness measures the extent to which a variable lies on the shortest path between other variables in the network. The negative value for Q6.1, Q6.5, Q6.11, and Q6.16 indicates that these items were less important in connecting other items in the network. On the other hand, the higher positive value for Q6.4, Q6.19, and Q6.20 suggests that these items played a crucial role in connecting other items in the network.

Closeness measures the extent to which a variable is close to other variables in the network. Higher values for closeness indicate that the item is more closely connected to other items in the network. In this table, Q6.4, Q6.19, and Q6.20 had the highest closeness values, indicating that these items were the most central in the network.

Strength measures the sum of the weights of the edges connected to a variable. Higher values of strength indicate that the item is more strongly connected to other items in the network. Results show that Q6.4, Q6.19, and Q6.3 had the highest strength values, indicating that these items were the most important in the network. In terms of node strength and the total of edges connecting to a node, the most important items were item 19 (I am ambitious) (betweenness weight of 1.852), item 4 (I have a high degree of intellectual capacity) (strength weight of 2.139), and item 13 (I have great faith in my own ideas and my own initiative) (closeness weight of 1.036).

Expected influence estimates the expected impact of a variable on other variables in the network. Higher values for expected influence indicate that the item has a greater expected impact on the other items in the network. In this table, Q6.4 and Q6.19 had high expected influence values, indicating that these items had a significant impact on other items in the network. Likewise, expected influence, the sum of a node’s connections representing the relative importance of a node in a network [51], was highest for item 4 (I have a high degree of intellectual capacity) (expected influence weight of 1.933) and item 13 (I have great faith in my own ideas and my own initiative) (expected influence weight of 1.263).

Overall, the results suggest that items Q6.4, Q6.19, and Q6.20 were the most central and influential items in the network. These items may be important indicators of adaptive overt narcissism.

In sum, item 4 (I have a high degree of intellectual capacity) had the strongest edge, the greatest node strength, and the highest predicted effect, indicating that it was the symptom most connected to other symptoms in the adaptive overt narcissism network.

In terms of negative expected influence, i.e., the potential nodes expected to deactivate the AOND network, we obtained the following results: the most negative expected influence was observed for item 16 (I am individualistic) (expected influence weight of −1.834), closely followed by item 8 (I appreciate art and beauty) (expected influence weight of −1.596). This suggests that responses to this item were associated with lower scores on other items in the AONS scale, indicating that individuals who scored high on Q6.8 tended to score lower on other items in the scale. Within the context of the AONS scale, individuals who strongly agree with this statement may be less likely to influence the overall network structure compared to individuals who do not strongly agree with this statement. As depicted in the literature review, the personal individualistic aspect is highly culturally influenced; hence, we take into consideration the second item, item 8 (I appreciate art and beauty), which represents, in our opinion, a self-elevating coping mechanism. Results, thus, validate our hypothesis. In addition to a strong cultural influence, the contemplation of art and beauty, seen as a self-elevating coping mechanism, has the most potential to deactivate the adaptive overt narcissism network.

Overall, the centrality measures depicted in Table 2 can provide insights into the structure and relationships among the items in the AONS scale, as well as help identify key items that may have a particularly strong influence on the overall score.

It is important to note that expected influence is a relative measure, and a negative score does not necessarily indicate that the item is unimportant or negatively valued in the context of the network. Rather, it indicates that this item may be less central to the overall structure of the network, or less likely to play a crucial role in transmitting information or affecting the behavior of other individuals in the network.

In terms of the specific item Q6.8, it may be interesting to explore why this item had a negative expected influence score. One possible explanation is that individuals who strongly agree with this statement may be more focused on their own aesthetic preferences and less interested in conforming to the preferences or values of others in the network. Alternatively, it could be that this item is less relevant to the specific social interactions captured by the AONS scale and, therefore, has less impact on the overall network structure.

Among the items that exhibited high centrality in the adaptive overt narcissism network, item 4 (I have a high degree of intellectual capacity) emerged as the symptom most connected to other symptoms. This item reflects the importance of intellectual abilities and self-perceived intellectual superiority within the construct of overt narcissism. Individuals who strongly endorse this statement may exhibit a strong need for intellectual validation and recognition, often seeking to showcase their cognitive prowess and considering themselves intellectually superior to others. Their self-esteem and self-worth may heavily depend on their perceived intellectual capabilities, leading to a heightened emphasis on intellectual achievements and an elevated sense of entitlement. Furthermore, item 13 (I have great faith in my own ideas and my own initiative) also displayed significant centrality in the network. This item captures the self-confidence and unwavering belief in one’s own ideas and initiatives that are commonly associated with overt narcissism. Individuals who strongly agree with this statement tend to exhibit a strong sense of self-assurance, viewing their ideas as inherently valuable and deserving of recognition. They may be highly driven by their own ambitions and less inclined to seek external validation or consider alternative perspectives. The high centrality of these items suggests their crucial role in the network structure, indicating that they significantly contribute to the manifestation of adaptive overt narcissism.

Further research could explore these possibilities, as well as the broader implications of the centrality measures presented in the table for understanding the dynamics of adaptive overt narcissism.

**Figure 2:**
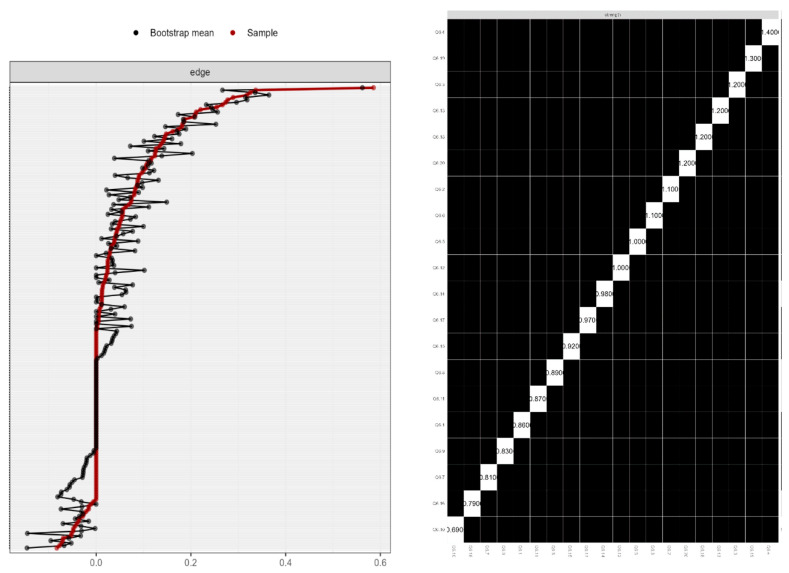
Network centrality stability.

Furthermore, using the case-dropping bootstrapping procedure, centrality stability tests were conducted (CS). Figure 2 displays the stability results for the sample’s global network. After monitoring data, the CS coefficient was used to evaluate the centrality indices’ stability. The results show a sign of network centrality stability.

The bootstrapped edge weights for each paired node comparison are shown in Figure 2 (left). The bootstrap mean is shown by the black line, point estimates for each edge weight are shown by the red line, and the edge weight 95% confidence intervals are shown by the gray shading.

Figure 2 (right) shows the bootstrapped centrality difference test. In the global AONS network, the bootstrapped significance of centrality estimations were evaluated for each paired node comparison (α = 0.05). Black boxes indicate significant differences, while the diagonal boxes represent the node strength values.

The stability centrality coefficient (CSC), which measures the proportion of instances that may be eliminated while still guaranteeing a correlation on the entire sample, gives this information. The guideline states that results from this study, with a CSC of 0.75, are robust.

## 5. Discussion

The current study examined the global network structure of the adaptive overt narcissism scale using a sizable and powerful sample dataset. It then evaluated whether an individual’s appreciation of art and beauty is a key factor in deactivating the adaptive overt narcissism network. Art and beauty appreciation proved to be the second most important factor in deactivating the adaptive overt narcissism network, after individualistic characteristic, according to the global network of the adaptive overt narcissism scale.

A recent study conducted in Germany found that being raised in a collectivist environment appears to lower narcissism and boost self-esteem [68]. These findings offer empirical proof that differences in narcissism and self-esteem are correlated with sociocultural factors. Narcissistic expressions have been more prevalent in individualistic societies. Individualistic cultures encourage a greater focus on the ego, whereas collective cultures place more weight on the importance of collective values. Individualistic culture members may be more narcissistic than collectivistic culture members because narcissism is characterized by a strong concentration on the self, a strong desire for praise, and extravagant illusions. Although our participants were from Romania, a country with its own unique cultural background, we believe that exploring the influence of art and beauty appreciation on the deactivation of adaptive overt narcissism can provide valuable insights beyond a single cultural context. By referencing the study from Germany, we aimed to highlight the broader relevance and potential universality of our findings. Regarding our results, we obtained a similar picture of the subclinical adaptive overt narcissist, which, in order to decrease the negative psychological pressure from investing too much energy to protect from the outside world, would need more individualistic traits to help them better preserve their status quo.

The second most important factor in deactivating the adaptive overt narcissist network is represented, as expected, by art and beauty appreciation. By placing an aesthetic value outside of the self, and through the admiration of something that is regarded as outside beauty, one can deactivate the general overt narcissist network. The contemplation of exterior beauty lowers the inflated image of the overt narcissist, but does not harm their status quo, mainly due to the fact that the inflated delusion of grandeur is presented to protect from the outside world. Grandiose, attention-seeking, entitlement, and an inflated sense of self (qualities that are typically anticipated in narcissists) are what define overt narcissists. Overt narcissists frequently exhibit high self-esteem and extraversion, but covert narcissists frequently exhibit low self-esteem, which can lead to defensiveness, insecurities, and self-consciousness. We might expect that art and beauty contemplation will do the opposite for the covert narcissistic personalities, which will feel threatened by beauty other than their selves.

Similar to our research, other narcissism scales have also referred to self-elevation behaviors, e.g., how the participant feels about art (e.g., “I do not like art”) in the Performative Refinement to Soothe Insecurities about Sophistication (PRISN) scale [69]. Furthermore, rather than true grandiosity and grandeur, narcissism is best viewed as a compensatory adaptation to overcome and cover up poor self-worth [69]. However, experts point out that not everyone with significant anxieties, such as narcissistic personalities, develops a self-elevating coping mechanism such as appreciating beauty and the arts. These results, therefore, agree with earlier data. Moreover, the network stability study demonstrated the robustness of the majority of the individually estimated networks. Our findings from subclinical populations across nations should be replicated in future studies. The robustness of upcoming comparisons would be increased by using bigger, better-matched sample sizes.

Art and beauty appreciation, in our situation, was the second most significant node after individualization in terms of predicted effect on the total network. As these nodes are most likely to activate other nodes, they may serve as targets for intervention to create a self-elevating coping style. Hence, less central but more flexible and plastic nodes may have a larger capacity for change and function as catalysts in the network for change in other nodes. With this type of psychotherapy, the adaptive overt narcissistic personality’s negative effects could be lessened.

Narcissists seldom acknowledge that their behaviors are problematic and may even deny doing so because they perceive it as a threat to their identity. Because of this, treating narcissism can be challenging. Vulnerability, reciprocity, and the ability to reflect are necessary for therapy. Art and beauty appreciation as a type of self-elevating coping style may be more tempting than other forms of treatment since those are precisely the things that we may define as dreadful, unpleasant, or difficult for someone with narcissism to accomplish.

The network analysis of the AONS scale provides valuable insight into the structure and relationships of the items in the scale. The centrality measures, such as betweenness, closeness, and strength, offer a way to identify the most important items in the network, and the expected influence measure helps us understand how much each item contributes to the overall structure of the network.

The results show that item Q6.4 (“I am aware of the influence I have on others”) had the highest betweenness and strength centrality measures, indicating that this item is the most important in the network. This finding is consistent with previous studies that have found that individuals with narcissistic traits are often aware of their ability to influence others [70,71]

Item Q6.9 (“I tend to exploit others towards my own goals”) had a positive betweenness centrality measure, indicating that it plays an important role in connecting other items in the network. This result is in line with previous research that has found that individuals with high levels of overt narcissism tend to manipulate and exploit others for their own benefit [72,73].

On the other hand, item Q6.8 (“I appreciate art and beauty”) had a negative expected influence measure, which suggests that this item has a weak influence on the overall structure of the network. This finding is consistent with previous studies that have found that narcissistic individuals tend to focus more on their own appearance and achievements rather than appreciating the beauty of others or the world around them [74,75].

Another study [76] found that grandiose narcissists, who exhibit an inflated sense of self-importance, are more likely than non-narcissists to regard themselves as possessing superior aesthetic taste. These studies support the idea that individuals high in narcissism may have a heightened appreciation for art and beauty, which aligns with the high strength score and negative expected influence score observed for item referring to art and beauty appreciation.

The network analysis results of the adaptive overt narcissism scale provide valuable insights into the psychological characteristics and relationships among the items. The centrality measures highlight key items that play a crucial role in the manifestation of adaptive overt narcissism. Item 4 (“I have a high degree of intellectual capacity”) emerged as the most connected symptom in the network. This suggests that individuals who strongly endorse this statement may exhibit a strong need for intellectual validation and recognition. Their self-esteem and self-worth may heavily depend on their perceived intellectual capabilities, leading to a heightened emphasis on intellectual achievements and an elevated sense of entitlement. Similarly, item 13 (“I have great faith in my own ideas and my own initiative”) also displayed significant centrality. Individuals who strongly agree with this statement exhibit a strong sense of self-assurance, viewing their ideas as inherently valuable and deserving of recognition. They may be highly driven by their own ambitions and less inclined to seek external validation or consider alternative perspectives. These findings indicate the importance of intellectual prowess and self-confidence within the construct of overt narcissism.

Furthermore, the negative expected influence score of item 8 (“I appreciate art and beauty”) raises interesting psychological considerations. Individuals who strongly agree with this statement may prioritize their own aesthetic preferences and be less concerned with conforming to the preferences or values of others in the network. It is possible that this item is less relevant to the specific social interactions captured by the AONS scale and, therefore, has less impact on the overall network structure. This suggests that the appreciation of art and beauty may not strongly contribute to the manifestation of adaptive overt narcissism as compared to other items in the scale.

The overall network architecture indicates that items reflecting dominance, resilience, intellectual capacity, and self-confidence are strongly interconnected. These findings align with previous research on overt narcissism and provide support for the network approach to understanding psychopathology symptoms. By examining the structure and relationships among the items, this analysis offers insights into the core components of adaptive overt narcissism and the dynamics of its manifestation.

It is important to note that the network analysis results do not replace the need for psychological analysis and interpretation. While these findings shed light on the interconnectedness of symptoms, it is crucial to consider individual psychological processes, subjective experiences, and contextual factors in understanding the complex nature of adaptive overt narcissism. Further research can delve deeper into the psychological mechanisms underlying the relationships among these symptoms and explore their implications for clinical assessment and intervention strategies.

The results of the network analysis provide a deeper understanding of the relationships among the items in the AONS scale and offer insights into the underlying structure of overt narcissism. These findings have implications for future research on narcissism and may inform the development of interventions aimed at reducing narcissistic behaviors.

Overall, our findings provide empirical support for the hypothesis that art and the appreciation of beauty can weaken the connections within the network of overt narcissism, leading to reduced levels of narcissistic behavior and attitudes. These results underscore the adaptive potential of engaging with art and highlight the importance of considering aesthetic experiences as a means to promote psychological wellbeing and enhance interpersonal relationships.

## 6. Conclusions

In conclusion, the network analysis of the AONS scale provided valuable insights into the centrality measures of the items, which could help to identify the most important nodes in the network. The results suggest that some items, such as Q6.4 (“I have a strong desire for admiration”), Q6.9 (“I feel better when others recognize my accomplishments”), and Q6.19 (“I know that I am special because everyone keeps telling me so”), play a more central role in the network, indicating that they are more strongly related to other items in the scale.

Interestingly, item Q6.8 (“I appreciate art and beauty”) had relatively low centrality measures in the network, suggesting that it is not as strongly related to other items in the scale. However, this item may still be important in the context of adaptive overt narcissism, as previous research has suggested that a focus on external beauty and image can be a key feature of narcissism [70].

Moreover, research has also shown that individuals high in narcissism may use art and beauty as a way to enhance their own self-image and status [70]. Therefore, it is possible that appreciation of art and beauty can also be a way for adaptive overt narcissists to gain admiration and attention from others.

Overall, these findings suggest that, while appreciation of art and beauty may not be a central component of the adaptive overt narcissist network, it still may play an important role in the expression and reinforcement of narcissistic tendencies. Future research could further explore the relationship between narcissism and art/beauty appreciation to better understand this dynamic.

Considering the large sample size of 1000 participants, our study had sufficient statistical power to detect significant relationships and patterns in the data. Therefore, we acknowledge that our sample size may not have been a significant limitation for this study. In terms of limitations, the study relied solely on self-reported data, which may have been subject to biases or social desirability effects. Lastly, the cross-sectional nature of the study design precluded any causal inferences about the relationships observed among variables. Further research with larger and more diverse samples, and with different methods of data collection and analysis, is needed to validate and extend these findings.

The results have clinical potential consequences for measuring adaptive overt narcissism and open up possibilities for creating future interventions that focus on encouraging individuals to appreciate beauty and the arts in order to destabilize the adaptive overt narcissism network in subclinical populations. Controlled studies are required to assess the effectiveness of such network-driven tailored therapies in lowering the overt narcissism network using art and beauty appreciations as a form of psychotherapy in cross-cultural studies and in clinical population samples.

## Figures and Tables

**Table 1:** Descriptive statistics for the Adaptive Overt Narcissism Scale (AONS).

Variable	Item	Mean	SD	Correlation Coefficient Q6.8	
1. Q6.1	1. I value my own independence and autonomy.	3.920	1.030	0.295	***
2. Q6.2	2. I set big goals for myself.	3.728	1.031	0.334	***
3. Q6.3	3. I have a wide range of interests.	3.823	0.923	0.390	***
4. Q6.4	4. I have a high degree of intellectual capacity.	3.635	0.909	0.331	***
5. Q6.5	5. I tend to have an unconventional way of thinking.	3.223	1.142	0.210	***
6. Q6.6	6. I genuinely value intellectual and cognitive matters.	3.665	0.998	0.347	***
7. Q6.7	7. I am verbally fluent and can express ideas well.	3.649	0.979	0.340	***
8. Q6.8	8. I appreciate art and beauty.	4.000	0.993	—	
9. Q6.9	9. I tend to be submissive, more of a follower than a leader. (R)	3.264	1.192	−0.126	***
10. Q6.10	10. I give up or even withdraw in the face of frustration and adversity. (R)	3.230	1.157	−0.108	***
11. Q6.11	11. My friends follow my lead.	3.071	0.954	0.210	***
12. Q6.12	12. I’m witty and charming with others.	3.466	0.976	0.277	***
13. Q6.13	13. I have great faith in my own ideas and my own initiative.	3.754	0.927	0.283	***
14. Q6.14	14. I am resourceful.	3.804	0.896	0.288	***
15. Q6.15	15. I am persevering.	3.762	0.924	0.328	***
16. Q6.16	16. I am individualistic.	2.972	1.146	0.015	
17. Q6.17	17. I am clever.	3.654	0.874	0.227	***
18. Q6.18	18. I am outgoing.	4.009	0.882	0.256	***
19. Q6.19	19. I am ambitious.	3.860	0.942	0.204	***
20. Q6.20	20. I am self-confident.	3.794	0.973	0.135	***

Note. *** *p* < 0.001.

**Table 2:** Centrality measures per variable.

Variable	Network			
	Betweenness	Closeness	Strength	Expected Influence
Q6.1	−1.189	−0.732	−0.833	0.040
Q6.2	0.223	0.648	0.485	1.148
Q6.3	0.440	0.793	1.096	0.409
Q6.4	1.852	1.025	2.139	1.933
Q6.5	−1.189	−0.530	−0.010	−0.866
Q6.6	0.548	0.137	0.339	0.560
Q6.7	−0.646	−0.174	−1.063	−0.153
Q6.8	−0.103	0.104	−0.644	−1.596
Q6.9	0.331	−2.256	−0.996	−0.998
Q6.10	−0.972	−2.458	−1.668	−1.349
Q6.11	−1.189	−0.358	−0.754	−0.894
Q6.12	−0.755	−0.065	−0.078	−0.139
Q6.13	0.548	1.036	1.040	1.263
Q6.14	−0.103	0.725	−0.202	0.571
Q6.15	−0.429	0.314	−0.527	0.297
Q6.16	−1.189	−0.966	−1.156	−1.834
Q6.17	0.657	0.534	−0.233	0.545
Q6.18	−0.320	0.388	0.873	−0.156
Q6.19	2.069	0.992	1.503	0.793
Q6.20	1.417	0.844	0.690	0.425

## Data Availability

The raw data supporting the conclusion of this study are available from the authors without restriction.
